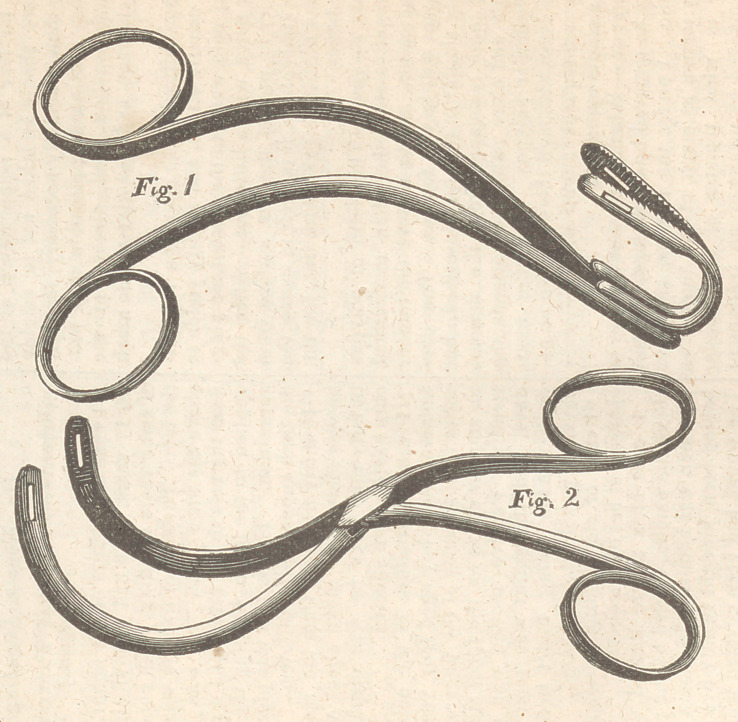# Jefferson Medical College

**Published:** 1843-06-24

**Authors:** H. T. Child


### CLINICAL LECTURES AND REPORTS.

### JEFFERSON MEDICAL COLLEGE.

### CLINIC OF PROFESSOR MÜTTER.

*Dispensary of Jefferson Medical College*, *Jan.* 25, 1843.

(Reported by H. T. Child.)

### LECTURE V.

Case I.—*Polypus of the Nose.*

(Continued.)

*Malignant Polypi.*—Malignant polypi of the nose assume different forms in their commencement, although they all produce nearly the same results before the case terminates. The term *malignant* is applied to those tumours, which, while by their increase they displace and destroy the bones and soft parts in their vicinity, also contaminate, or convert them into a substance similar to their own. It is almost impossible to detect the exciting causes of such growths; often they commence as simple benign polypi, and gradually degenerate; sometimes they appear malignant from the first; again, they may occasionally be traced to some cause apparently local; while often they seem to be dependent upon constitutional or hereditary predisposition. The duration of these cases is also very variable; in some the disease runs its course in a few months, and in others, years elapse before death comes to the relief of the sufferer. When the disease is strictly malignant from its commencement, it usually assumes one of three types; it begins either as *cancer of the mucous membrane*, *medullary sarcoma*, or it is *schirrous.*

Dr. Watson, of New York, in an excellent article on nasal polypi, published in the American Journal of Medical Sciences for 1842, describes another malignant type under the name of *gelatinous polypus.* But as this term might lead surgeons to confound a very simple and benign disease, with one that is most terrible in its results, and as from the description given of the tumour by Dr. Watson, I am disposed to consider it one of medullary sarcoma, I shall restrict my remarks to the description of the disease as it usually occurs.

1*st. Cancer of Mucous Membrane.*—This form is confined almost exclusively to old persons, and is characterized by the developement upon the mucous membrane, or rather by a conversion of this tissue into a red, vascular, soft and spongy growth, somewhat similar in appearance to the venereal wart. The pain is often severe, and of the peculiar kind belonging to cancer elsewhere, but sometimes there is scarcely any suffering until near the close of the case. As the tumour increases in size it projects into the antrum, forces through the cribriform plate of the ethmoid bone, destroys the vomer, and may ultimately attack the cheek. The secretion is acrid, sanious and alkaline, and often excoriates the parts over which it passes. Finally, the constitution gives way, and death closes the scene.

2*d. Medullary, or Fungous Polypus.*—This form of polypus may originate in the mucous membrane of the nose, but usually it begins in the antrum, or upper maxillary bone, and gradually extends into the nostril. It is often difficult to distinguish the malignant tumour, in its commencement, from a common fibrous or fleshy one, but the former is usually softer and redder, and rougher upon its surface; it also bleeds much more readily; the matter secreted is sanious and fœtid; it usually increases rapidly; the surrounding parts more speedily become involved: there is also pain of a peculiar kind; and the general health is sooner effected. I have known a case of this kind, however, remain firm and hard like a fibrous polypus, even to the last, and it was only by dissection that the medullary matter could be detected, but, generally, it runs the course of medullary or hæmatoid disease in any part of the body.

3*d. Schirrous Polypus.*—Another malignant tumour is occasionally met with in the nostril. It is firm, inelastic, shining, whitish, or slightly reddish, and generally, though not always, painful. When cut into it presents the gristly substance of ordinary schirrus and if neglected will run through the usual changes of this tissue, becoming softer, and ultimately throwing out a fungous growth. It of course will give rise to all the terrible symptoms of malignant polypus.

*External Polypi.*—The tumours described all occupy the cavity of the nostril at first, although as they increase in size, they may protrude and become external. But by *external* polypi we mean those tumours which originate from the integuments of the nose. They are met with among bon vivants, whose complexions indicate their propensities, and are usually located on the alæ nasi, or just above the lobe; they may, however, occupy any portion of the organ.

The disease consists in the enlargement of the sebaceous follicles, or the tumour may be composed of the whole integument in a state of hypertrophy. Usually the mass is of slow growth, scarcely sensitive, assumes a pyriform shape, and becomes pendulous, is bluish in colour, and may acquire an immense size.

It is often confounded with lipoma, or cancer of the nose, but differs essentially from these affections, and has no tendency to become malignant.

*Diagnosis.*—When a polypus really exists there is usually not much difficulty in recognizing its presence, but it often happens that both the patient and surgeon are led to suppose that a tumour has formed in the nostril, when in reality there is nothing of the kind. When a person is brought to you, therefore, a careful examination should be made ere you say that he has or has not a polypus. To examine the part the patient must be seated opposite a strong light, and the head so placed that the rays may fall into the nostril; often this is sufficient to enable you to detect the polypus, which appears as a shining mass, whitish, reddish, or yellowish, as the case may be, and fills up the nostril, more or less, completely. But even when the tumour is obvious, we must ascertain its consistence, the location of its root, and its mobility; to do this, the little finger should be oiled and carried carefully into the nostril. Where this is too small to admit the finger, or the patient is a child, we must ascertain the character of the tumour, by passing a probe around its base; we may also make the person blow forcibly through the nose, which will cause the tumour, if moveable, to change its position. Sometimes the tumour is small, and located far back in the nostril; then the anterior nares must be distended either with a speculum or a pair of common dressing forceps, the blades of which being introduced into the nostril and expanded, serve the purpose of a speculum very well. When the disease is seated in the posterior portion of the nasal cavity, and points towards the fauces, we ascertain its position and character by passing the forefinger behind the velum, and into the posterior nares. Pushing the tumour back with a director and making the patient inhale his breath forcibly, will materially assist in the examination of such cases.

From the fact that most of the symptoms of polypus are those which would be occasioned by any obstruction in the nostril, it is not surprising that mistakes in diagnosis are so frequently made, even when the examination has been carefully conducted. The affections with which it is most frequently confounded, are

1st. An unusual size of the inferior turbinated bone.

2d. An inclination of the septum narium to one side.

3d. Disease of the bones of the nose.

4th. Œdema of the mucous membrane from cold.

5th. Chronic thickening of the mucous membrane from inflammation, (as in scrofula, dyspepsia, &c.)

6th. Abscesses.

7th. Ozena, (followed by ulcers and granulations.)

8th. Fibrous tumours, or cysts in the submucous cellular tissue—which differ from vesicular polypi.

9th. Polypus of the antrum.

10th. Hernia cerebri.

11th. Foregn bodies lodged in the nostril.

Malformation of the inferior turbinated bone is probably the defect most frequently mistaken for polypus, but we can generally distinguish the former by its *hardness, peculiar shape, extensive attachments, and by its location being lower down*, or nearer the floor of the nostril than the genuine polypus.

I have known it, however, mistaken for the disease, and the whole bone, or a large portion of it torn away by the forceps under this impression.

Inclination of the septum to one side can usually be recognized by the *shape of the septum* ; by *one nostril being larger than the other;* and by the fact that the finger or probe *passes in readily on one side, and is arrested by a firm substance almost at the entrance of the other*.

Dieffenbach, Velpeau, Watson, and myself, and probably others, have met with this difficulty, and I have repeated the operation of Dieffenbach for its relief wi h manifest benefit to my patient.

Caries, necrosis, osteo-sarcoma, or exostosis can generally be detected by referring to the history of the case. Venereal, or scrofulous disease, is the usual cause of caries and necrosis of the bones of the nose, but they may result from blows, and previous inflammation of the lining membrane. The discharge in such cases is free and often most disagreeable and fœtid.

Œdema of the Schneiderian membrane is recognized by its *extended base, peculiar cause*, (cold for example,) and the rapidity with which it usually makes its appearance after exposure to whatever brings it on. If neglected, it will lay the foundation of either vesicular or gelatinous polypi.

Chronic inflammation, followed as it usually is by thickening of the pituitary membrane, and submucous cellular tissue, may be distinguished by the history of the case, the peculiar redness of the membrane, and by the absence of every thing like a distinct tumour. We may also derive assistance in making the diagnosis, by passing a probe along the nostril. When chronic inflammation exists, it is introduced without difficulty, but in polypus the point is arrested by the tumour. Dyspepsia and scrofula are the most common causes of this affection.

Acute inflammation terminating in abscess has been mistaken for polypus, but here the *history of the case*, the *peculiar pain*, and *finally fluctuation in the swelling*, should at once indicate the nature of the difficulty. Where the abscess opens and granulations sprout from the ulcer that remains, and fill up the nostril, as in the case of Mr. Keate's patient, referred to by Hawkins, there is more difficulty in forming a diagnosis.

Ozena, dependent upon any cause, is often mistaken for polypus; but in ozena, at least in its early stages before ulceration and granulation have taken place, we can always pass a probe from one end of the nostril to the other without difficulty; there is little or no tumour, and the secretion is also very fœtid and peculiar.

Fibrous, encysted, and other tumours have been mistaken for polypus, but there is no difficulty in arriving at a correct diagnosis, if we are careful in examining the structure and location of the swelling; they are, of course, of different consistences, and generally spring from the septum.

The protrusion of a portion of a polypus of the antrum into the nostril, is often supposed to be an example of na‿al polypus, but in these cases we have for the most part, though not invariably, the upper jaw enlarged and painful, and often we are enabled to detect the neck of the tumour as it emerges from the opening of the antrum.

Cruveilhier and others have reported a case or two of hernia cerebri, where the tumour by descending into the nostril through the foraminæ in the cribriform plate was mistaken for polypus.

Finally, the introduction and retention of foreign bodies in the nostril, have given rise to all the symptoms of polypus. Peas, buttons, beads, &c., have been removed from patients who have supposed themselves labouring under the complaint in question.

*Prognosis.*—This will depend entirely upon the character and extent of the disease; when the tumour is *benign, of recent formation, not very large*, or *firm*, the *parts* in its *vicinity sound*, and *the patient's general health not affected*, there is usually not much difficulty in removing every vestige of the disease. But when the reverse of this is the case, when, for example, the tumour is *malignant in its nature from the first*, or when it *assumes this character in consequence of neglect;* when it is large and firm, and *adheres closely to the parts around it;* when the *bones of the nose and face are forced out of their position* or *become involved in the disease, and when the patient*

*is pale and sallow,* with a depraved condition of his digestive apparatus, the prognosis is highly unfavourable, and very few cases should be touched by the surgeon.

*Treatment.*—Few subjects in surgery have been more laboriously or ingeniously handled than the treatment of nasal polypi; but it would be worse than useless to waste time in a description of the various measures, some of them harsh and dangerous, devised by different surgeons. I shall, therefore, describe as briefly as possible, the treatment proven by experience to be the best adapted to each form of the complaint.

1st. *Vesicular Polypus.*—This variety being, as I have already stated, usually connected with a depraved condition of the general system, it becomes our duty to resort, whenever such is the case, to constitutional as well as local measures for its cure. If we find the tongue furred, the bowels costive or too loose, the liver torpid, the appetite poor, the nervous system depressed or excited—when, in short, there exists evident derangement of the chylopoietic viscera it will be proper to administer the blue pill in alterative doses, with some of the simple bitters or alkalies, to regulate the diet, to use the warm bath occasionally, and, if possible, to resort to one of the sulphurous mineral springs. I have also in these cases derived benefit from the nitro-muriatic acid exhibited both internally and as a pediluvium after the manner of Mr. Scott.

When there is evidently an anemic condition existing, or where the usual characteristics of a strumous diathesis are present, the greatest confidence may be placed in an alterative course of iodide of potassium with sarsaparilla.

The local treatment here consists, first, in the breaking up, and partial or complete removal of the tumours; and secondly, in the application of the different vegetable and mineral astringents, by which their regeneration will be prevented.

To remove the tumours, the patient should be seated in a good light, with his head supported by an assistant, and the upper part of his body covered with a sheet to receive the blood. The surgeon then seats himself in front, and begins the operation by slowly and carefully introducing up the nostril the blades of a pair of straight fenestrated polypus forceps, until he reaches the base or pedicle of the tumour. He then expands the blades and seizes the diseased mass, as near its attachment as possible, then, after one or two slight movements, to ascertain that the instrument is properly applied, he closes the blades firmly; and finally, by giving the forceps a rotary motion, he gradually twists off the tumour, which is brought away in the grasp of the blades. This done, he requests the patient to blow forcibly through the nostril, and if the operation is successful there is no difficulty in his doing this, but should a portion of the tumour remain, or should there be others still attached, no air passes, and he feels himself as much "stuffed up" as ever. The forceps are then to be reintroduced again and again, until the obstruction is completely removed—at least this should be done if the patient can bear it,—but often from previous irritation or pain, or faintness, we are obliged to suspend our efforts and postpone to another occasion, the completion of the operation. When the tumours are inaccessible tc the straight forceps, one with a slight curve will sometimes answer a very good purpose. And when they are situated far back and overhang the fauces I have often employed others, of the shape represented in the annexed figures. When either of these is used it must be carried through the mouth, down in-

Fig. 1
Fig. 2

to the pharynx, and then drawn up behind the velum so that the blades may embrace the tumour, which is detached by rotating the instrument, and may then be withdrawn through the mouth.

In the vesicular polypus, however, I have rarely had the good fortune to bring away the tumour entire, for, from its delicacy, the first grasp of the forceps usually causes its rupture, and when the instrument is withdrawn, it contains but a few shreds of cellular membrane. When this is the case we must endeavour to break up the mass as effectually as possible, and then rely upon astringents for the removal of what remains, and of these agents I prefer the sulphate of copper to all others. This salt may be used in solution, (ʒj. to f℥viij. of water,) and to apply it properly it should be thrown into the nostril by means of a syringe; the usual practice of snuffing up a liquid is very inefficient, as it is impossible for the patient to draw it into the upper portion of the nostril. When the tumour has been removed, and there remains a disposition in the membrane to form others, or to throw out granulations; I have derived great benefit from the use of a snuff made of the root of the sanguinaria canadensis, and my friend, Dr. Thomas Harris tells me that he has seen an excellent effect produced by powdered rhubarb, used in the same manner. The snuff is taken several times a day. Finally, where there exists a tendency to chronic inflammation of the Schneiderian membrane, or ozena, there is nothing to be compared for permanent efficacy with the inhalations of mercury, and I employ them in the following manner. Take of cinnabar, grs. xx., white sugar, grs. lx., mix and divide in twenty powders, one of these is to be placed upon a piece of iron previously heated, though not to redness—over which a small glass funnel is inverted, as soon as the fumes of the powder are disengaged. By introducing the end of the tube into the nostril, the fumes will be made to penetrate every portion of the cavity, and thus act upon the entire surface of the diseased membrane. These remedies must be used for some time after the nostrils seem clear, for fear of a reappearance of the disease. But care should be taken not to employ them any longer than is actually necessary, for their repeated and long continued application may produce chronic inflammation, and disease of the Schneiderian membrane, and thus give rise to much mischief.

The hæmorrhage following the operation for vesicular polypus is usually slight, and readily yields to the application of cold water to the nostril, or plugging the anterior nares with a little lint. After the operation, in order to protect the raw surface from the action of the atmosphere, it is well to cause the patient to keep the nostril plugged with lint, for the first twenty-four hours.

2d. *Gelatinous Polypus.*—This form of polypus rarely, if ever, requires any other than mechanical means for its eradication, and to remove the tumour the best instrument will be the forceps. The operation is precisely similar to that just described, but we may expect more resistance from the tumour, more hæmorrh ge and more pain, than attends the removal of a vesicular polypus. The after treatment is also the same, although there is often no necessity for any subsequent attention, the disease being cured by a single operation.

3d. *Fleshy Polypus.*—When the tumour belongs to this class it may be removed by the forceps, ligature, scissors or caustic. I prefer the forceps, and after getting away by torsion as much of the tumour as possible, apply to what remains of the pedicle, lunar caustic, conveyed by means of an instrument similar to the porte caustique of Lallemand. In some cases when the tumour is readily reached, and is attached by a long pedicle of no great diameter, it answers very well to remove it with the scissors or knife, and then touch its base with caustic, but the hæmorrhage is generally profuse, and although we can arrest it by ice, styptics, plugging the nostril, &c. &c., yet it often occasions difficulty, and we should never undertake the operation without being fully prepared for any emergency that may arise from this cause. When the tumour is large, and has a broad or extended pedicle, and there exists in the patient the hemorrhagic diathesis, it will be safest to apply the wire ligature as near the base as possible, and then remove the body of the tumour with the scissors. By this method we avoid the risk of hæmorrhage, and also save our patient the disagreeable accompaniment of an extensive sloughing of the whole mass. When it is impossible, from the location of the tumour, to reach it with the scissors, it must be left to separate by sloughing, and during this process the nostril must be frequently syringed out with carrot water, or creosote water, or solution of chloride of lime, or pyroligneous acid and water, in order to correct the fetor and render the patient comfortable.

Should the loop of the ligature become loose it must be daily tightened, by drawing upon the wire and giving it an additional turn around the wing of the canula. After the detachment of the tumour the use of the sanguinaria canadensis in powder as a snuff is often very beneficial.

(To be continued.)

## Figures and Tables

**Fig.1 Fig.2 f1:**